# Treatments with versus without medication for children with behavioural difficulties in clinical practice: an economic evaluation with observational data

**DOI:** 10.1111/jcpp.14057

**Published:** 2024-09-30

**Authors:** Caitlin K. Kiernan, Hermien H. Dijk, Barbara J. van den Hoofdakker, Pieter J. Hoekstra, Annabeth P. Groenman

**Affiliations:** ^1^ Department of Child and Adolescent Psychiatry University of Groningen, University Medical Center Groningen Groningen The Netherlands; ^2^ Accare Child Study Center Groningen The Netherlands; ^3^ Department of Economics, Econometrics, and Finance University of Groningen Groningen The Netherlands; ^4^ Department of Clinical Psychology and Experimental Psychopathology University of Groningen Groningen The Netherlands; ^5^ Research Institute Child Development and Education (RICDE) University of Amsterdam Amsterdam The Netherlands

**Keywords:** Economic evaluation, pharmacotherapy, behaviour problems, ADHD, longitudinal studies

## Abstract

**Background:**

Economic evaluations of treatments for children with behavioural difficulties (i.e., characteristics of attention‐deficit/hyperactivity disorder (ADHD) and/or oppositional defiant disorder (ODD)) usually rely on data of randomised controlled trials or are model‐based. Findings of such studies may not be representative of cost‐effectiveness and cost‐utility in clinical practice. The current longitudinal study aimed to perform an economic evaluation of treatments for children with hyperactivity, impulsive behaviours, inattention, and/or behavioural difficulties using observational data that were obtained in clinical practice.

**Methods:**

Parents of 209 children (aged 5–12) who were referred to 1 of 10 Dutch youth mental healthcare institutions and who received treatment with (*n* = 108) or without (*n* = 101) the use of medication, filled out questionnaires at three timepoints (baseline, and ~ 6 and ~12 months later). Propensity score matching was used to make both groups comparable. Outcomes included quality‐adjusted life years (QALYs), ADHD and ODD symptom severity, and impairment. Costs were measured from a societal perspective. Incremental cost‐effectiveness ratios (ICERs) were estimated, and cost‐effectiveness acceptability curves (CEACs) were derived to show uncertainty around the ICER.

**Results:**

Results did not show statistically significant differences in costs and effects between children who were treated with medication (alone or in combination with non‐medication treatment) and those who were treated without medication. CEAC suggested that medication treatment has a 55% probability of being cost‐effective at the €80,000 threshold and 36% at the €20,000 threshold compared with treatment without medication.

**Conclusions:**

Using observational data, our study did not provide clear evidence of the cost‐effectiveness and cost‐utility of treatment with medication compared with treatment without medication in clinical practice.

## Introduction

Attention‐deficit/hyperactivity disorder (ADHD) and behavioural difficulties are prevalent in children and adolescents worldwide (Polanczyk, Salum, Sugaya, Caye, & Rohde, 2015) and associated with high societal costs (Le et al., 2014), as well as adverse educational (Galéra, Melchior, Chastang, Bouvard, & Fombonne, 2009) and occupational outcomes (Klein et al., 2012). Evidence suggests that treatment of ADHD can prevent or mitigate some of the adverse long‐term outcomes that are associated with ADHD (Franke et al., 2018) and thus reduce the lifetime costs associated with the disorder.

Treatments for ADHD in children include pharmacological treatment (stimulants or non‐stimulants) and non‐pharmacological treatment (such as behavioural parent training) (National Institute for Health and Care Excellence [NICE], 2019). From the perspective of policymakers, the costs of treatments to the healthcare system and society as a whole are essential aspects of the treatment decision, especially since healthcare funds are limited (Raad voor de Volksgezondheid & Zorg, 2007) and policymakers have the objective to maximise health given the available funds (Brouwer, van Baal, van Exel, & Versteegh, 2019).

Decisions regarding the allocation of funds can be informed by economic evaluations, such as cost‐effectiveness analyses (CEAs) or cost‐utility analyses (CUAs). CEAs compare alternative treatments in terms of costs and (clinical) outcomes, whilst CUAs do so in terms of costs and utility, where utility is the benefit of treatment in terms of (usually) quality‐adjusted life years (Drummond, Sculpher, Claxton, Stoddart, & Torrance, 2015). The results of CEAs and CUAs are generally summarised in an Incremental cost‐effectiveness ratio (ICER) or an Incremental cost‐utility ratio (ICUR), respectively. An ICER or ICUR is the difference in costs between the alternative treatments divided by the difference in outcomes (i.e., effectiveness or utility measures, depending on whether the study is a CEA or CUA). The ICER or ICUR can be interpreted as the additional costs of an extra point effect or extra QALY gained, respectively (Drummond et al., 2015). Guidelines for conducting these types of economic evaluations have been written by, among others, the National Institute for Health and Care Excellence (NICE, 2022) and the Dutch National Health Care Institute (2016).

Previous economic evaluations of ADHD treatments for children have been based on randomised controlled trials (RCTs) or were model‐based, such as evaluations based on Markov models and decision trees (Dijk et al., 2021). The current literature on the cost‐effectiveness of ADHD treatments (see Dijk et al., 2021 and Sampaio, Feldman, Lavelle, & Skokauskas, 2021, for reviews) provides indications that stimulant and non‐stimulant pharmacological treatments and parent training can be considered cost‐effective treatments for children compared with no treatment (Dijk et al., 2021). Previous trial‐based cost‐effectiveness studies comparing medication and behavioural treatments for children with ADHD found that medication management was likely to be cost‐effective compared with combination treatment and behavioural treatment for ADHD without comorbidities (Foster et al., 2007; Jensen et al., 2005). However, the cost‐effectiveness of treatments for children with ADHD with and without comorbidities might differ (Foster et al., 2007). Previous model‐based studies focused on medication treatment rather than non‐medication treatments (see Dijk et al., 2021). There have also been trial‐based studies that examined the cost‐effectiveness of ADHD treatments outside the clinical setting, such as the study by Margherio, Evans, Monopoli, and Langberg (2023) evaluating a school‐based training for adolescents with ADHD.

Both types of studies have important benefits. For instance, RCT‐based studies have high internal validity resulting from randomisation of treatment and control groups (Drummond, 1998). Model‐based studies have the benefit of allowing to combine available evidence from a variety of sources (Petrou & Gray, 2011) and extrapolate the costs and effects of treatments over longer periods of time (Weinstein, 2012). RCTs are often considered the gold standard, but they are generally performed under highly controlled (ideal) circumstances (Drummond, 1998; Singal, Higgins, & Waljee, 2014) or do not compare all treatments that are being used in clinical practice (Petrou & Gray, 2011). Therefore, their generalisability to the cost‐effectiveness of treatments in clinical practice may be limited.

If we aim to inform policy makers about the allocation of limited healthcare funds, we also need to have evidence of the cost‐effectiveness of treatments in clinical practice. This can be done using observational data. Although observational data may be subject to selection bias as a result of non‐randomisation into treatment (Angrist & Pischke, 2009), a variety of advanced statistical techniques exist that allow the estimation of treatment effects and which have previously been used in the context of economic evaluations, such as covariate matching, propensity score methods, instrumental variables, and difference‐in‐differences (see Rovithis, 2013).

Economic evaluations comparing the wide variety and combinations of treatments that children with behavioural difficulties receive in clinical practice have not yet been performed using observational data. As such, the cost‐effectiveness of treatments for children with behavioural difficulties in clinical practice remains unknown. In the current study, we performed an economic evaluation using longitudinal observational data from clinical practice on children between the ages of 5–12 with behavioural difficulties (i.e., characteristics of ADHD and/or oppositional defiant behaviour). We aimed to examine the cost‐effectiveness and cost‐utility of treatment with medication compared with treatment without medication during a 1‐year time horizon from a societal perspective. Since we used observational data, the treatment and control groups were not randomised. It could be that some children who received treatment with medication differed at baseline from those who did not and were therefore more likely to be pharmacologically treated. For instance, symptom severity could be higher for those who were offered treatment with medication compared with those who were not. We addressed this possible selection into treatment by using propensity score matching.

## Methods

### Participants and procedure

In the Netherlands, children can be referred to youth mental healthcare institutions by their general practitioner, medical specialist, or municipality (e.g., through a municipal youth team). Parents and children do not face any out‐of‐pocket costs for youth mental healthcare after referral. Participants were recruited at a total of 10 youth mental healthcare institutions with various locations across the Netherlands that perform diagnostics for ADHD and offer pharmacological and behavioural parent treatment.

We aimed to include participants before they received any treatment, therefore, a specific diagnosis was not a requirement for participation in the study. Instead, inclusion in the study was based on whether parents reported that their child was referred to a youth mental healthcare institution with inattention, hyperactivity, impulsivity, or behavioural problems, rather than a specific diagnosis.

With their invitation for the first appointment at the youth mental healthcare institution, parents received information about the study and were able to give written informed consent. After consent, caregivers were invited via email to fill out online questionnaires about their referred child. The first questionnaires (T0) were sent as soon as possible after the child had been referred and before a diagnosis or treatment decision was made. The second (T1) and third (T2) sets of questionnaires were sent to parents ~ 6 months after T0 and ~ 6 months after T1, respectively. Parents received a gift card after completing each questionnaire. Data collection took place between 2017 and 2020. Observations with missing values in all variables (i.e., 44 at T0, 24 at T1, and 16 at T2) were removed from the dataset, as the observations were completely empty and these parents therefore did not actually participate in that respective survey. Parents were included in the analysis if they had participated in all three surveys.

### Ethics

The study was performed in accordance with the ethical standards as laid down in the 1964 Declaration of Helsinki and its later amendment in 2013.

### Comparator treatments

Since we used observational data, children were not randomised into treatments. Treatment and reference groups were constructed based on questionnaire responses from parents at T1 and/or T2 regarding treatments initiated since the start of the care trajectory. We created a ‘treatment group’ which consisted of children who had initiated medication treatment only or medication combined with other non‐medication treatments. The comparator group consisted of children whose parents did not indicate medication treatment initiation during the care trajectory. We will refer to these groups as treatment with medication and treatment without medication groups, respectively.

### Costs

The Intensive Youth Care Questionnaire (*Vragenlijst Intensieve Jeugdzorg*, Bouwmans et al., 2012) was used at T0 and T2 to collect information on the utilisation of healthcare services and support services in other sectors by both the parent and child during the previous 3 months, as well as productivity loss of the parent during the previous month. An example item of the questionnaire is: *Has your child had contact with the care providers listed below in the past three months in relation to psychological, emotional, or behavioural problems? If so, how often?*

A detailed description of the calculation of all cost components can be found in Appendix S1. Consumption of healthcare services and support services in other sectors were multiplied by the reference prices provided in the Cost Manual of the Dutch National Health Care Institute (Hakkaart‐van Roijen, van der Linden, Bouwmans, Kanters, & Swan Tan, 2016), the Intensive Youth Care Questionnaire manual (Bouwmans et al., 2012), or the Intersectoral Costs and Benefits manual (Drost, Paulus, Ruwaard, & Evers, 2014). Costs of medication were constructed using prices provided on the website Medicijnkosten.nl (medication costs; Dutch National Health Care Institute (2022), last accessed on September 15, 2022). Only costs of medication were included, costs related to picking up prescriptions were not assessed. Costs related to travel to and from providers were not included in the cost estimation, as we did not have information on the distance to providers and modes of transportation to each of the services considered in this study. Productivity losses for paid work were calculated using the friction‐cost method and the average hourly wage in the Netherlands (Hakkaart‐van Roijen et al., 2016). For unpaid work, parents were asked how many hours others (i.e., family members, other unpaid persons, home care, and other paid persons) had to take over housekeeping tasks due to the behavioural difficulties of their child. The total number of hours was multiplied by the reference prices for productivity loss of unpaid work (Hakkaart‐van Roijen et al., 2016). Opportunity costs for parents' and children's leisure time were not included in the analyses, as there is, to the best of our knowledge, no consensus on how to value this. Monetary valuations of leisure time can be considered arbitrary and can lead to a double‐count (Hakkaart‐van Roijen et al., 2016). All reference prices were indexed for the year 2020 using the Consumer Price Index (CBS, 2021). A detailed description of the calculation of all cost components can be found in Appendix S1 and an overview of the reference prices used to calculate costs can be found in Table S1.

### Utility and effectiveness

Cost‐utility analyses (CUA) compare alternative treatments in terms of incremental costs and utilities, where utility can be measured using quality‐adjusted life years. Cost‐effectiveness analyses (CEA) do so in terms of incremental costs and outcomes, where outcomes might be classification‐specific.

Utility was assessed using quality‐adjusted life years (QALYs) obtained with the adult Dutch version of the EuroQol‐5D‐5L (EQ‐5D‐5L) at T0 and T2 (EuroQol Research Foundation, 2019). Parents were asked to rate their child's health‐related quality of life in five dimensions: mobility, self‐care, daily activities, pain, and anxiety/depression on a five‐level categorical scale. EQ‐5D‐5L responses were calculated into QALYs using the Dutch EQ‐5D‐5L tariff for adults (Versteegh et al., 2016), as a tariff for Dutch children is not yet available.

Since the items included in the EQ‐5D may not be relevant for children with externalising behaviours (e.g., the pain domain), as the questions do not reflect externalising or social problems (Mierau et al., 2020), and treatment is focused on improving impairment (Fabiano & Pelham, 2002) and reducing symptoms of behavioural difficulties, we also used ADHD and oppositional defiant disorder (ODD) symptom severity and impairment as outcome measures in the CEA.

The Swanson, Nolan, and Pelham Rating Scale (SNAP‐IV, Swanson et al., 2001) was used as a measure of ADHD and ODD symptom severity. The 18 items for hyperactivity‐impulsivity and inattention were combined into an average score ranging from 0 to 3, where a higher score indicates higher ADHD symptom severity. A score of equal or less than 1 indicates that a child has low symptom severity (Swanson et al., 2001). Similarly, in a separate variable, the 8 items for ODD were combined into an average score ranging from 0 to 3 for ODD symptom severity.

Impairment was measured using the Impairment Rating Scale (IRS, Fabiano et al., 2006). The IRS asks parents to rate the severity of their child's impairment in seven domains: (1) relationship with peers, (2) relationship with siblings, (3) relationship with parents, (4) academic progress, (5) self‐esteem, (6) family functioning, and (7) overall impairment. Parents indicated their child's impairment on a visual analogue scale ranging from 0 (indicating *no problems*) to 100 (indicating *severe problems*) in each domain. In line with Fabiano et al. (2006), the line was then divided into seven equally spaced segments ranging from 0 (indicating *no problems*) to 6 (indicating *severe problems*). Individual item scores were combined into an average score ranging from 0 to 6, where a higher score indicates increased parent‐perceived impairment.

We used Cronbach's alpha to measure the reliability of the outcome measures in our raw data. All clinical instruments showed good reliability, whilst the EQ‐5D showed poor reliability.

### Analysis

All analyses were performed in Stata SE 17.0 (StataCorp, 2021). In order to handle missing data, we employed multiple imputation using chained equations (MICE, White, Royston, & Wood, 2010) with predictive mean matching and used five nearest neighbours to draw from. The following variables were imputed: age of child and parent, ADHD and ODD symptom severity at T0 and impairment, EQ‐5D index, health care costs of child and parent, other services costs of child and parent, medication, absenteeism, presenteeism, and unpaid work costs at T0 and T2. An overview of frequencies and percentages of missing variables can be found in Table S2 in Appendix S2. The following variables were included in the imputation model: mental health of the parent at T0 and T1 (measured using the Mental Health Inventory‐5), impairment at T1, ADHD and ODD symptom severity at T1 and T2, and sex of the child. Values were imputed by the comparator group, meaning that imputation was performed in the treatment with medication and treatment without medication groups separately. We performed 25 imputations.

After imputation, the cost components were summed into two categories: cost due to productivity loss and costs for healthcare and other services (both parents and child). Subsequently, log transformation was applied to the two cost variables (i.e., $\ln C=\ln\;\left(C+1\right)$) to deal with the long right‐tail of the cost components.^1^

In order to obtain an estimate for the outcome variables of interest, we use the ‘within’ approach, as recommended by Granger, Sergeant, and Lunt (2019), meaning that after multiple imputation, we compute a propensity score matching estimate in each imputed dataset, and combine them by taking the average estimates of all imputed datasets.

In each imputed dataset, a logit model was used to calculate linear predictions of the propensity scores, that is, the probability of receiving treatment with medication. Austin (2011b) recommends including variables in the estimation of the propensity score that determines treatment assignment. Since sex and symptom severity are significant predictors of medication recommendation for children with ADHD (Courtabessis et al., 2018; Russell, Ford, & Russell, 2019), sex of the child, ADHD and ODD symptom severity at baseline were included in the propensity score model. Furthermore, impairment, QALY's, log healthcare costs, log costs due to productivity loss, and age of the child at baseline were included. Log costs at baseline were included as high costs might be an indication that children already received care before intake, which might have influenced their probability of receiving treatment with medication. Additionally, high costs might be an indication that there are other possible influences or circumstances that might lead to higher costs at T2, but that are not directly related to treatment for behavioural problems.

After the estimation of the propensity score, a matched sample was created using the user‐written Stata command *gmatch* ^2^ (Lunt, n.d.), which performs greedy matching. This means that it selects matches based on the proximity of the propensity score and without replacement. We used a caliper distance of 0.2 times a standard deviation, as recommended by Austin (2011a), in order to ensure that similar individuals are matched. Summary statistics and standard errors were derived using Rubin's rule (Granger et al., 2019; Rubin, 1987). Standardised differences^3^ in each imputed dataset were averaged to obtain a single standardised difference. A standardised difference of 0.10 and greater is said to indicate the imbalance between the groups (Austin, 2009, 2011b). We further examined variances of continuous variables in the comparator groups and variance ratios, as recommended by Austin (2009).

The estimates for the log of resource utilisation and the log of productivity loss costs were transformed back to level costs.^4^ These were then linearly interpolated to span the entire 12‐month period between surveys, assuming that the effect of treatment on resource utilisation and productivity loss is 0 at baseline and increases linearly over time. After interpolation, estimated incremental resource utilisation and productivity loss were combined into a single cost estimate.

Bootstrapping was used to account for the uncertainty introduced by multiple imputation and propensity score matching. Previous studies on bootstrapping and multiple imputation (without the extra step of using propensity score matching) provide conflicting suggestions on which of these steps to bootstrap (Bartlett & Hughes, 2020; Schomaker & Heumann, 2018). Literature on how to compute confidence intervals using bootstrapping when combining multiple imputation and propensity score matching is scarce.^5^ Therefore, we combined one of the methods proposed by Schomaker and Heumann (2018) with propensity score matching by drawing 400 bootstrap samples from each of the imputed datasets and estimating outcomes in each bootstrap sample using propensity score matching. The 95‐percentile confidence intervals were constructed by ordering the 25 × 400 = 10,000 bootstrap estimates.

### Cost‐utility and cost‐effectiveness analysis

We compared children who, according to questionnaire responses from their parents, initiated treatment with medication (i.e., either medication only or medication combined with other non‐medication treatments) to children who did not report medication initiation during the care trajectory. The estimated incremental effect of pharmacological treatment on QALY's, ADHD and ODD symptom severity, and impairment were combined with the interpolated effects on costs into four incremental cost‐effectiveness ratios (ICERs), one for each outcome measure (i.e., QALYs, ADHD and ODD symptom severity, and impairment). The ICER can be interpreted as the costs of an additional QALY or unit decrease in effect measure (i.e., ADHD symptom severity, ODD symptom severity, and impairment).

To show the statistical uncertainty surrounding the ICER, we generated cost‐effectiveness planes (CE‐planes) and we derived a cost‐effectiveness acceptability curve (CEAC) to illustrate the probability that treatment with medication is cost‐effective compared with treatment without medication against a maximum willingness to pay for a QALY. The CE‐planes were derived by combining the bootstrap results for costs and effects from each imputed dataset (i.e., 25 × 400 = 10,000 replications), which were then plotted against each other. The CEAC was derived by bootstrapping with 400 replications in each imputed dataset and combining the resulting ICERs into a single dataset consisting of 10,000 ICERs (i.e., 25×400=10,000 replications), which were sorted in ascending order and plotted as a CEAC up to a threshold of €80,000 per QALY, as is the suggested threshold by the Council for Public Health and Health Care (Raad voor de Volksgezondheid & Zorg, 2007) and the highest of three thresholds suggested by the Dutch National Health Care Institute (2015). The latter advises thresholds of €20,000, €50,000, and €80,000 for low, intermediate, and high disease burden, respectively, where ADHD and conduct disorders would qualify as having low disease burden (Salomon et al., 2015).

### Sensitivity analyses

We performed 10 sensitivity analyses to verify the robustness of our results to assumptions about costs, decisions regarding the sample, and propensity score method. A detailed description of each sensitivity analysis can be found in Appendix S3 and Table S3 contains alternative references prices used in some of the sensitivity analyses.

## Results

### Sample and descriptive statistics

Since not all questionnaires were filled out by two parents for all children, we used only questionnaires completed by the primary parent. Our sample consisted of 368 children, of whom 209 parents (56.8%) completed all three surveys. We compared the baseline characteristics of those children to those whose parents had either (partially) completed survey T0 only (*n* = 113, 30.7%) or survey T0 and T1 only (*n* = 35, 9.5%) using Welch's t‐tests and Chi‐squared tests.^6^ The results can be found in Table S4A,B in Appendix S4. Children for whom not all three surveys were completed had significantly lower ADHD symptom severity. We calculated age using the reported birth date and the date of the baseline survey. Ten parents reported birth dates for their children and themselves resulting in an unlikely calculated age (e.g., 1 or −1). In these cases, age was set to missing.

The treatment with medication group (i.e., initiated medication only or medication combined with other non‐medication treatments during the care trajectory) consisted of 108 children, whilst parents of 101 children did not report medication initiation during the care trajectory.^7^ Children in the treatment with medication group often also received other non‐medication treatments in addition to medication. An overview of the non‐medication treatments that were reported by each treatment group can be found in Table S5 in Appendix S5.^8^

Table 1 shows the characteristics of children with and without medication treatment at baseline, pre‐ and post‐imputation, and propensity score matching. Prior to propensity score matching, standardised mean differences for nearly all variables exceed 0.1, which is indicative of an imbalance between the comparator groups.

**Table 1:** Baseline descriptive statistics pre‐ and post‐imputation and matching

Variables	Raw data^a^			Multiply imputed and matched data		
	Treatment with medication	Treatment without medication	Standardised mean differences^b^	Treatment with medication	Treatment without medication	Standardised mean differences^b^
	Mean (*SD*) or frequency (%)	Mean (*SD*) or frequency (%)		Mean^b^ (*SE*)^c^	Mean^b^ (*SE*)^c^	
Age of child	8.33 (1.66)	8.24 (1.63)	0.05	8.32 (0.45)	8.25 (0.45)	−0.04
Sex of child (female)	25 (23.1%)	35 (34.7%)	**0.26**	18.5	18.8	0.07
Treatment history (yes)	39 (36.1%)	13 (12.9%)	**0.56**	24.9	10.8	**0.47**
ADHD symptom severity	1.76 (0.56)	1.43 (0.64)	**0.54**	1.63 (0.26)	1.63 (0.26)	0.00
ODD symptom severity	1.26 (0.70)	1.31 (0.80)	0.07	1.26 (0.29)	1.30 (0.30)	0.05
Average impairment	3.40 (1.13)	3.13 (1.41)	**0.21**	3.33 (0.37)	3.34 (0.40)	0.01
EQ‐5D‐5L index	0.77 (0.16)	0.80 (0.18)	**0.16**	0.78 (0.14)	0.79 (0.15)	0.06
Age of caregiver	38.33 (5.71)	37.59 (6.26)	**0.13**	37.97 (0.90)	37.55 (0.94)	−0.07
MHI‐5 caregiver	73.63 (14.90)	71.25 (18.12)	**0.14**	73.68 (1.65)	79.89 (1.68)	**−0.22**
ln (health and other service cost)	6.11 (1.54)	5.26 (2.23)	**0.44**	5.81 (0.46)	5.91 (0.47)	0.06
ln (productivity loss cost)	0.88 (2.28)	0.70 (2.00)	0.08	0.79 (0.52)	0.92 (0.54)	0.06

Treatment with medication: children who reported medication treatment initiation alone or in combination with non‐medication treatments. Treatment without medication: children who did not report medication treatment initiation. Standardised mean differences greater than 0.10 are bold. ADHD, Attention‐Deficit/Hyperactivity Disorder; EQ‐5D‐5L, EuroQol‐5D‐5L; MHI‐5, Mental Health Inventory 5; ODD, Oppositional Defiant Disorder; *SD*, Standard deviation; *SE*, standard errors.

^a^ Includes missing values.

^b^ Average standardised mean differences of 25 imputed datasets. The number of matched pairs, and therefore the sample size, differs per imputed dataset, with an average of 71.08 matches. The minimum number of matched pairs in an imputed dataset was 69 (total sample size 138) and the maximum 73 (total sample size 146).

^c^ Standard errors calculated using Rubin's rule.

In propensity score matching, an average of 71.08 matches (i.e., matched treatment with medication and treatment without medication pairs) were made in the 25 imputed datasets, with the minimum number of matches being 69 and the maximum 73, meaning that our sample size reduced from 209 subjects to a range of 138–146 subjects after matching. After multiple imputation and propensity score matching, the standardised mean differences of the child‐related variables, with the exception of treatment history, were all less than 0.1, which is an indication that the groups are fairly balanced when it comes to these variables. Variables relating to the parent, that is, mental health, showed signs of imbalance. Table 2 presents the variance ratios between the groups in the raw data and the sample after imputation and matching. The variance ratios were closer to unity after imputation and matching.

**Table 2:** Variances of continuous variables pre‐ and post‐imputation and matching

Variables	Raw data			Multiply imputed and matched data		
	Variance treatment with medication	Variance treatment without medication	Ratio with medication to without medication variances	Variance treatment with medication	Variance treatment without medication	Ratio with medication to without medication variances
Age of child	2.74	2.66	1.03	0.21	0.20	1.02
ADHD symptom severity	0.31	0.41	0.75	0.07	0.07	0.93
ODD symptom severity	0.49	0.64	0.77	0.08	0.09	0.91
Average impairment	1.27	1.98	0.64	0.14	0.16	0.86
EQ‐5D‐5L index	0.03	0.03	0.84	0.02	0.02	0.85
Age of caregiver	32.61	39.13	0.83	0.80	0.88	0.91
MHI‐5 of caregiver	221.92	328.23	0.68	2.72	2.82	0.97
ln (health and other service costs)	2.38	4.98	0.48	0.22	0.22	0.97
ln (productivity loss costs)	5.18	4.01	1.29	0.27	0.29	0.94

Treatment with medication: children with reported medication treatment initiation alone or in combination with non‐medication treatments. Treatment without medication: children with no reported medication treatment initiation. Variance in the multiply imputed and matched sample is the total variance, i.e., $V_{w}+V_{B}+\frac{V_{B}}{25}$, where $V_{w}$ is within variance and $V_{B}$ is between variance.

### Cost‐effectiveness analysis

**Figure 1:**
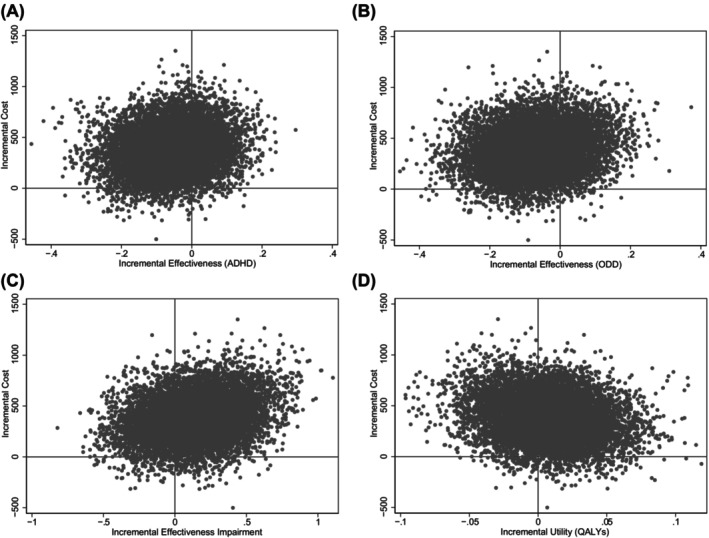
Cost‐effectiveness planes. The CE‐planes are based on 25 × 400 = 10,000 bootstrap replications

The results of the cost‐effectiveness analysis can be found in Table 3. We found higher QALY's, more costs, and impairment, but lower ADHD and ODD symptom severity in the treatment with medication group compared with the non‐pharmacological treatment group. However, zero was included in the 95%‐CIs for all incremental effect outcomes, indicating that we did not find statistically significant differences between treatment with medication and treatment without medication in terms of the effect on QALYs, ADHD and ODD symptom severity, impairment, or costs. To show the uncertainty surrounding the results of the cost‐effectiveness analysis, CE‐planes were derived. The CE‐plane comparing cost and our outcome measures can be found in Figure 1 and show that for all outcomes, the results are generally spread over the north‐eastern and north‐western quadrants, implying that treatment with medication is generally more costly and that there is uncertainty about its effectiveness in relation to treatment without medication.

**Table 3:** Results main analysis: treatment with medication compared with treatment without medication

Variables	Mean differences^a^	95% CI^b^
QALY	0.005	−0.048; 0.063
ADHD symptom severity	−0.057	−0.234; 0.113
ODD symptom severity	−0.100	−0.265; 0.127
Impairment	0.149	−0.342; 0.620
Total costs	389.23	−16.48; 809.23
ICER QALY	77,354.01	
ICER ADHD symptom severity	−6,779.43	
ICER ODD symptom severity	−3,876.39	
ICER impairment	2,617.53	

Treatment with medication: children with reported medication treatment initiation alone or in combination with non‐medication treatments. Treatment without medication: children with no reported medication treatment initiation. ADHD, Attention‐Deficit/Hyperactivity Disorder; ICER, incremental cost‐effectiveness ratio; ODD, Oppositional Defiant Disorder; QALY, quality‐adjusted life year.

^a^ Average mean differences of 25 imputed datasets. Costs are interpolated.

^b^ Percentile 95% CI's derived using bootstrap with 400 replications in each imputed dataset.

**Figure 2:**
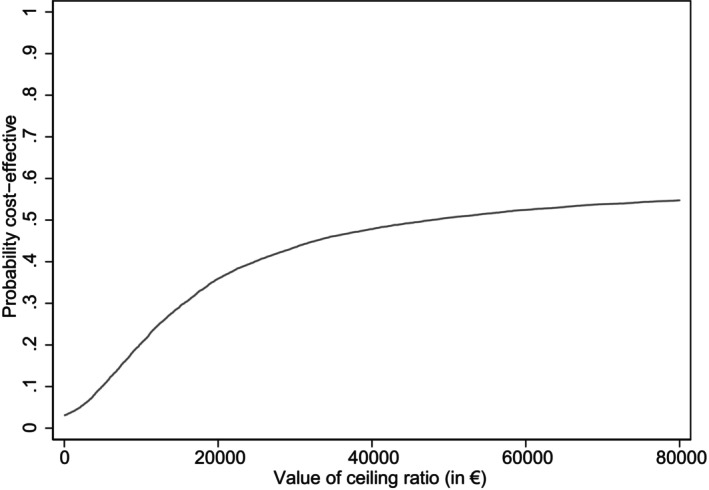
Cost‐effectiveness acceptability curve treatment with medication compared with treatment without medication. CEAC is based on 25 × 400 sorted bootstrapped replications. Treatment with medication: children with reported medication treatment initiation alone or in combination with non‐medication treatments. Treatment without medication: children with no reported medication treatment initiation

The ICERs that were found (€ 77,354.01 per QALY, € −6,779.43 per point ADHD severity, € −3,876.39 per point ODD severity, and € 2,617.53 per point reduction in impairment) should be evaluated against different willingness‐to‐pay thresholds. The CEAC for the ICER comparing cost and QALYs for treatment with medication and treatment without medication can be found in Figure 2. With a threshold of € 80,000 the CEAC demonstrates that there is a 55% probability that treatment with medication is cost‐effective compared with treatment without medication based on QALYs in a clinical practice setting. When a threshold of € 20,000 is applied, the CEAC shows a 36% probability of cost‐effectiveness.

### Sensitivity analyses

We performed 10 sensitivity analyses to verify the robustness of our results to assumptions about costs, decisions regarding the sample, and propensity score method. The results of the sensitivity analyses can be found in Appendix S6 and in Table S6. The sensitivity analyses generally show that our results are robust to changes to the sample when it comes to the incremental effectiveness outcomes. Incremental cost outcomes were sensitive to the exclusion of those who reported treatment history in the previous year and to an alternative propensity score method. Furthermore, incremental cost outcomes were sensitive to the addition of extra non‐medication treatment sessions to the costs of those who indicated the use of these treatments, as the incremental costs became larger and had 95%‐CIs that excluded zero. This may be due to higher percentages of reporting non‐medication treatments in the treatment with medication group.

## Discussion

The aim of this study was to examine the real‐life cost‐effectiveness and cost‐utility of common treatments for children aged 5–12 with behavioural difficulties. We used observational data from parents of children between the ages of 5–12 referred for behavioural difficulties to youth mental healthcare institutions in the Netherlands, which reflects clinical practice. In contrast to previous RCT‐based and model‐based economic evaluations, we observed treatments that were actually used in clinical practice. We used propensity score matching in order to deal with possible selection bias resulting from the use of observational data. We compared children who received treatment with medication to children who did not receive medication.

We found small and non‐significant differences between the two groups in mean QALYs, ADHD and ODD symptom severity scores, impairment scores, and costs. Furthermore, the results of our CEAC indicated that the probability of treatment with medication compared with not receiving medication being cost‐effective is 55% and 36% for thresholds of € 80,000 and € 20,000 per QALY, respectively. A comparison of the two treatment groups after propensity score matching showed that the groups were fairly balanced in the main variables of interest. However, it is possible that not all systematic differences were eliminated, hence, we must exercise caution in interpreting the results as causal.

This study adds to the existing literature on the cost‐effectiveness and cost‐utility of treatments for children with behavioural difficulties. Previous studies have consisted of RCT‐based and simulation model economic evaluations. Whilst these are informative, they do not always reflect clinical practice, thus limiting their generalisability to real‐life cost‐effectiveness. Therefore, it is important that economic evaluations using observational data are also performed.

It is difficult to directly compare our results to previous RCT‐based studies, such as those by Jensen et al. (2005) and Foster et al. (2007). These studies found that medication management was likely to be cost‐effective compared with combined treatment and behavioural treatment for ADHD without comorbidities (Foster et al., 2007; Jensen et al., 2005) for children with ADHD. However, the cost‐effectiveness of treatments for children with ADHD with and without comorbidities might differ (Foster et al., 2007). The differences between our results and those of previous studies might be the result of using different study designs, considering different treatment groups, samples, cost perspectives, and cost components, and conducting the studies in different healthcare contexts.

For instance, in RCT‐studies such as those of Jensen et al. (2005) and Foster et al. (2007), treatment was randomised and there was a research protocol which set out the exact treatment participants would receive. In clinical practice, this is not always the case. Whilst practitioners have clinical guidelines, recent studies (Dekkers et al., 2022; Matthijssen et al., 2022) concluded that practitioners do not always follow these guidelines. Also, guideline adherence may differ between settings, such as mental healthcare settings versus paediatric settings (Matthijssen et al., 2022). Furthermore, clinicians may base their treatment recommendations on their attitudes towards the treatment and prior clinical experience (Dekkers et al., 2022). Indeed, our data show that in clinical practice, children and their caregivers received a variety of treatments, both in the treatment with medication group as well as in the group with medication. Whilst children thus start various, and sometimes multiple, treatments in real‐life, this is often not reflected in the strict protocols of RCTs.

Furthermore, whilst previous studies focused on children with an ADHD diagnosis (including Foster et al., 2007; Jensen et al., 2005), our sample included children who were referred to youth mental healthcare institutions because of behavioural difficulties, but who were not necessarily diagnosed with ADHD. This led to a broad range of children in our study, which may be more reflective of clinical practice than the criteria for participants in studies using RCT data and could therefore be more informative for policy makers who need to make decisions regarding limited healthcare funds. Jensen et al. (2005) employed a societal perspective of costs and included the direct costs associated with the treatment. Foster et al. (2007) included the real costs of the treatments of the Multimodal Treatment Study of ADHD (MTA study). In our study, we employed a broader definition of the societal perspective, and in addition to the direct costs of treatment, also included indirect healthcare costs as well as costs due to productivity loss. Consideration of different cost components can lead to different assessments of cost‐effectiveness and cost‐utility.

Furthermore, the MTA study on which Jensen et al. (2005) and Foster et al. (2007) are based was undertaken in the United States. The Dutch healthcare system differs from that of the United States and the costs of healthcare differ in each system (Anderson, Hussey, & Petrosyan, 2019). These differences in costs could also lead to different cost‐effectiveness results.

Similarly, it is difficult to compare our results to those found in model‐based studies (such as those using a simulation model), as the number of studies that compare pharmacological treatment to non‐pharmacological treatments is low. Most studies compared various forms of pharmacological treatment or used no treatment as an alternative, whilst our study considered a wide variety of non‐pharmacological treatments as comparator. Freriks et al. (2019) compared pharmacological, behavioural, combination, and community treatment, using delinquency as an outcome measure, and found that community care was optimal. However, since they use a different outcome measure, it is not comparable to the outcome measures used in our study.

The current study faced several limitations. To deal with selection bias resulting from the use of observational data, we used propensity score matching. Whilst it is possible that not all systematic differences were eliminated (for instance, if unbalance remains in variables that were not observed), our sample showed balance in the main variables of interest. However, it should be noted that matching caused our sample to reduce from 209 subjects to a sample ranging between 138 and 146 subjects. This small sample size is a possible reason why we did not find significant results. Future studies examining the cost‐effectiveness of treatments for behavioural difficulties should aim to use larger samples.

Furthermore, only 57% of participants completed all measurements and those that were lost to attrition had significantly lower symptom severity scores compared with those that had completed all three questionnaires. This suggests that those with lower severity problems may not necessarily need treatment. Whilst attrition within our study's time horizon might be a clear representation of those families seen in clinical practice, it does limit the generalisability of our results. Another possible limitation relates to the way we measured costs in this study. We performed cost‐utility and cost‐effectiveness analyses from a societal perspective, where we included costs based on healthcare utilisation, service utilisation in other sectors, and costs based on productivity loss. However, we did not include travel costs of parents and children to these services, as we did not have this information, which could be a limitation of this study. Furthermore, we relied solely on parental reports in this study. For healthcare and other service utilisation, which was used to determine cost differences between treatment groups, it is unclear whether these reports provide an accurate reflection of actual utilisation. Future studies could incorporate administrative health records to derive the costs of treatments.

Since we only observed care utilisation and productivity loss in the last 3 months, we may not have observed a large part of the treatment period. We dealt with this by using linear interpolation to estimate the effect on costs for the entire 12‐month period and by performing sensitivity analyses in which we added various extra costs for non‐pharmacological treatments based on guidelines and clinical practice for those who had indicated that such a treatment had started (for individuals in the treatment with medication group as well as those in the treatment without medication group). Whilst it might still be possible that care utilisation in practice differs from our assumptions, our sensitivity analyses showed the robustness of our results to these assumptions.

We used the EQ‐5D to measure utility in the cost‐utility analysis and examined the reliability of this measure in our sample using Cronbach's alpha, which showed poor reliability. We used the value set for Dutch adults, since one for Dutch children is not yet available. The EQ‐5D might not accurately measure health‐related quality of life for children with behavioural problems, since it does not include questions on psychosocial problems (Mierau et al., 2020). However, the EQ‐5D is the preferred measure for health‐related quality of life according to guidelines for economic evaluations by the National Institute for Health and Care Excellence (NICE, 2022) and the Dutch National Health Care Institute (2016) and has the benefit that it allows for comparisons across studies and treatments. Future studies could explore cost‐utility of treatments for children with behavioural problems using instruments that also include questions in psychosocial domains or externalising behaviour.

The time horizon in this study was 1 year and this might be relatively short. Since treatments were still ongoing for several subjects, costs and effects used in this study might not be the complete image. These outcomes could be examined in future studies by using observational data covering a longer period, for instance from childhood into adulthood (Nagy et al., 2017). This would allow researchers to fully assess long‐term treatment effects in clinical practice.

## Conclusion

This cost‐utility and cost‐effectiveness study was the first to use data from clinical practice to compare common treatments for children who were registered with a youth mental healthcare institution with behavioural difficulties. Our results did not indicate any statistically significant differences between treatment with medication and treatment without medication in terms of QALYs, ADHD or ODD severity, impairment, and costs. The probability of treatment with medication being cost‐effective was 55% for the threshold of € 80,000 and 36% at the € 20,000 threshold. As our study was limited by small sample size, future studies with larger sample sizes are recommended to draw generalisable conclusions about the cost‐effectiveness of common treatments for children with behavioural difficulties in clinical practice. Despite these limitations, and in contrast to previous studies, our findings suggest that in clinical practice, treatment with medication may not be cost‐effective compared with treatment without medication when considering a 1‐year time horizon.

**Key points**

- Cost‐utility and cost‐effectiveness of treatments for children with behavioural problems have mostly been studied using data from randomised controlled trials or through modelling studies.
- We examined cost‐utility and cost‐effectiveness of common ADHD treatments using observational data of children between age 5 and 12 in the Netherlands.
- We found no significant differences in QALYs, symptom severity, impairment, or costs between those who reported treatment with medication and those who reported only treatment without medication.
- Future studies should assess cost‐utility and cost‐effectiveness using a larger sample and considering a time horizon that exceeds one year.

## Supporting information


**Appendix S1.** Description of calculation of costs.
**Appendix S2.** Missing data.
**Appendix S3.** Sensitivity analyses: methods.
**Appendix S4.** Attrition.
**Appendix S5.** Treatment combinations.
**Appendix S6.** Sensitivity analyses: results.
**Table S1.** Unit cost per resource and costs of productivity loss.
**Table S2.** Frequency and percentage missing.
**Table S3.** Assumed the number of sessions and reference prices in sensitivity analyses.
**Table S4.** Sample attrition: Comparison of baseline characteristics. (A) Questionnaire T0 and T1 only vs questionnaire T0, T1 and T2. (B) Questionnaire T0 only vs questionnaire T0, T1, and T2.
**Table S5.** Treatment combinations in raw data.
**Table S6.** Sensitivity analyses: interpolated costs and effects.

## Data Availability

Supporting data are not publicly available, as participants did not provide written consent for the public sharing of their data.

## Endnotes

1 Since there are many zeros in the cost variables, $\ln C=\ln\left(C+1\right)$, where *C* refers to the cost variables (i.e., health and other sector costs and productivity loss costs), was used rather than $\ln C=\ln\left(C\right)$.

2 The *gmatch* command was written by Mark Lunt. For more information on this command, see the Stata help file. This command does not have a version listed in its Stata help file. The last time this command was (re)installed for this study was between August 25 and August 27, 2023.

3 Standardised differences in each imputed dataset were derived using the user‐written command *stddiff*, written by Ahmed M. Bayoumi (distribution date: 2022‐12‐17).

4 In order to transform the log mean difference in cost found using propensity score matching ($\beta_{1}=\ln\left(C_{t}\right)-\ln\left(C_{u}\right)$) to level costs, we used the equation $Y=\exp\beta_{0}\left(\exp\beta_{1}-1\right)$, where $\beta_{0}$ is the constant, $\beta_{1}$ is the estimated mean difference, $C_{t}$ is the estimated mean in the treatment with medication group, $C_{u}$ is the estimated mean in the reference group and Y refers to the level mean difference in costs. Mean differences for health and other sector costs and productivity costs were estimated separately and combined after transforming back to level cost.

5 A recent study by Eiset and Frydenberg (2022) combined multiple imputation and propensity score weighting by imputing, estimating, and combining within each bootstrap replication, finding 95‐percentile confidence intervals with 999 replications.

6 The remaining parents had the following patterns: *n* = 6 (1.6%) had completed T0 and T2 only, *n* = 3 (0.8%) had completed T1 and T2 only, and *n* = 2 (0.5%) had completed T1 only.

7 One caregiver did not indicate that the child started medication treatment at T2, while they did indicate methylphenidate use at T2 for their child. This caregiver‐child pair was therefore allocated to the treatment with medication group. One caregiver indicated ‘X’ on all medication use at T2 but did not fill in any dosage information for any of the medications and did not indicate that medication treatment had started. The ‘X’ has therefore been interpreted as ‘no’ and the caregiver was not allocated to the treatment with medication group and costs for medication were set to zero instead of missing. For three caregivers treatment assignment is only based on the response at T1, as they submitted incomplete surveys at T2.

8
*N* = 35 parents indicated that none of the treatments listed in the questionnaire had been initiated during the care trajectory, although some did indicate that a treatment had been chosen. It is unclear whether these subjects actually did not receive any treatments, so they were included in the main analysis. We performed a sensitivity analysis in which we exclude these subjects from the sample.
