# Supplementary material for: Treatments with versus without medication for children with behavioural difficulties in clinical practice: an economic evaluation with observational data
Source: J Child Psychol Psychiatry. 2024 Sep 30;66(3):289–300. doi: 10.1111/jcpp.14057 (PMC11812490; doi:10.1111/jcpp.14057)
Supplement: Supplementary file 1 — Appendix S1. Description of calculation of costs. Appendix S2. Missing data. Appendix S3. Sensitivity analyses: methods. Appendix S4. Attrition. Appendix S5. Treatment combinations. Appendix S6. Sensitivity analyses: results. Table S1. Unit cost per resource and costs of productivity loss. Table S2. Frequency and percentage missing. Table S3. Assumed the number of sessions and reference prices in sensitivity analyses. Table S4. Sample attrition: Comparison of baseline characteristics. (A) Questionnaire T0 and T1 only vs questionnaire T0, T1 and T2. (B) Questionnaire T0 only vs questionnaire T0, T1, and T2. Table S5. Treatment combinations in raw data. Table S6. Sensitivity analyses: interpolated costs and effects. [file JCPP-66-289-s001.docx]

**Appendix for: Treatments with versus without medication for children with behavioural difficulties in clinical practice: an economic evaluation with observational data.**

Caitlin K. Kiernan^1, 2, 3^, Hermien H. Dijk^1,2,3^, Barbara J. van den Hoofdakker^1,2,4^, Pieter J. Hoekstra^1,2^, and Annabeth P. Groenman^1,2,5^

^1^ Department of Child and Adolescent Psychiatry, University of Groningen, University Medical Center Groningen, Groningen, the Netherlands

^2^ Accare Child Study Center, Groningen, The Netherlands

^3^ Department of Economics, Econometrics, and Finance, University of Groningen, Groningen, the Netherlands

^4^ Department of Clinical Psychology and Experimental Psychopathology, University of Groningen, Groningen, The Netherlands

^5^ Research Institute Child Development and Education (RICDE), University of Amsterdam, Amsterdam, the Netherlands

**Appendix S1. Description of calculation of costs**

The economic evaluation was performed from a societal perspective, meaning that costs both within and outside of the healthcare sector were considered, as well as productivity losses of the parent. The Intensive Youth Care Questionnaire (*Vragenlijst Intensieve Jeugdzorg*, Bouwmans et al., 2012) was used at T0 and T2 to collect information on the utilisation of healthcare services and support services in other sectors by both the parent and child during the previous three months, as well as productivity loss of the parent during the previous month.

For healthcare services and services in other sectors parents were asked to indicate whether the child and the parent themselves used the service in the previous three months and, if so, how often they and the child used the service. When parents replied with answers such as “daily”, “weekly”, or “monthly”, service utilisation was calculated based on the assumption that a 3-month period consists of 90 days or 12 weeks. When parents indicated that a service was used for examination or referral purposes, it was assumed that the service was used once. If parents indicated that a service was used “at least” x times, or “more than” x times, it was assumed that the service was used x times. When parents’ replies were unclear (e.g., “a couple of times”, “many”, or “often”, or a range) or when they indicated that they could not remember, service utilisation was set to missing, as service utilisation could not be inferred from the reply. If parents indicated in the parent items that they used a service with/for their child, parent service use was considered zero for the specific item in order to avoid overlap with the child service use item.

Consumption of healthcare services and services in other sectors were multiplied by the reference prices provided in the Cost Manual of the Dutch National Health Care Institute (Hakkaart-van Roijen et al., 2016), the Intensive Youth Care Questionnaire manual (Bouwmans et al., 2012), or the Intersectoral Costs and Benefits manual (Drost et al., 2014). An overview of the reference prices used to calculate costs and their source can be found in Table S1. All reference prices were indexed for the year 2020 using the Consumer Price Index (CBS, 2021). If parents indicated that a healthcare service or support service were not used, it was assumed that costs for those variables were zero. Individual cost components were summed in the following cost components: total healthcare costs child (excluding medication costs), total costs other services child, total healthcare costs parent, and total costs other services parents. If any of the individual cost components was missing for a parent-child pair, the aggregated cost component was also considered missing and values were imputed in the multiple imputation stage prior to analysis.

For the questions about use of babysitter, pre-/between-/after-school day care, medical day care and Boddeart center, parents were asked to indicate how many quarter days the care was used. From the responses it became clear that many interpreted this as hours, or the exact time the care was used could not be derived from the answer. Therefore, we assumed for all reported numbers that it referred to number of hours rather than quarter days, which could be an underestimation in some cases.

There were several instances where items were not included in the calculation of the cost components. First, parents were asked about their child’s truancy. According to the Intensive Youthcare Questionnaire manual (Bouwmans et al., 2012), costs due to truancy only result if a child has to repeat a year of school. As the current study has a time horizon of 1 year and we do not know whether children had to repeat years of school, we cannot include costs due to truancy in total cost. Moreover, parents were asked to indicate how much time a week they and their child spent on exercises, these were not used to calculate total cost as it is unclear what the exercises consisted of and how to express these in terms of costs. Additionally, parents were asked if their child had contact with other workers, however, individuals either answered only the type of worker without frequency of contact or frequency of contact but not the type of worker, therefore, this category was also not used to calculate total cost. Furthermore, parents were asked about utilisation of community centres and religious institutions (specifically, Church, Mosque or Synagogue). However, since there is no reference cost for such services, they were not included in the calculation of aggregated cost components. In each of the four previously listed instances, items were dropped from analysis. Consequently, if parent-child pairs had a missing in only one of these items (and not in any other health or other service items) their aggregated cost component was not considered missing and thus not imputed.

In order to determine cost of medication, parents were asked whether their child used any of the following medications during the previous three months: methylphenidate (specifically, Ritalin, Concerta, Equasym, Medikinet, and methylphenidate were each listed separately), dexamphetamine, atomoxetine (specifically Strattera), dipiperon, pipamperon, or risperidone (Risperdal, Risperdal Consta^^[[1]](#footnote-1)^^). If so, they were asked to provide information on dosage. If parents indicated that a medication was used but did not respond dosage or the information on dosage was unclear (e.g., if parents responded that they did not know dose or frequency), dosage for that medication, and therefore costs, were set to missing. If a parent indicated that the child received a different dose during different days of the week, an average dose per day was calculated. If a parent indicated that the child only sometimes received an extra dose, but did not specify frequency of that extra dose, only the usual dosage was used. As the recall period of the questionnaire is 3 months, it was assumed that if parents indicated that a medication was used (almost) daily or for 7 days a week, that the medication was used for 90 days in total, and if they indicated that a medication was used only on schooldays, that the medication was used for 60 days in total (assuming that there are 5 schooldays per week and 20 schooldays per month). If medication was missing and the child was not part of the treatment with medication group, costs were set to zero rather than missing so that these values would not be imputed for those who did not use medication.

Using dosage information, costs of medication were constructed using prices provided on the website Medicijnkosten.nl (Dutch Institute National Health Care, last accessed on September 15, 2022). A reference price per milligram was calculated for each of the medications, which were then multiplied by dose, frequency per day, and number of days in the previous 3 months ^^[[2]](#footnote-2)^^. Possible costs of issuing medications by pharmacies have not been included in the calculation of costs for medication. An overview of the reference prices used to calculate costs and their source can be found in Table S1. All reference prices were indexed for the year 2020 using the Consumer Price Index (CBS, 2021).

Productivity losses for paid work were calculated using the friction-cost method, which assumes that employees who are absent for long durations (i.e., exceeding 85 calendar days) will be replaced by someone who was previously unemployed, or who themselves is replaced by someone who was previously unemployed (Koopmanschap et al., 1995). Absenteeism was measured by asking the primary parent how many days of work they had missed in the past month because of the behavioural difficulties of their child. If this exceeded a month, they were asked what the date of their first day of sick leave was. In this case, absenteeism was derived by calculating the number of missed days based on the date on which the survey was completed. When work missed was less than 85 days, productivity losses due to absenteeism were calculated by multiplying missed days with the average number of hours per workday and the productivity cost per hour, where the average hours per workday is derived by dividing the reported number of working hours per week by the reported number of workdays a week. If absence from work exceeded 85 days, then only the productivity losses in the first 85 calendar days since the start of the absenteeism were computed. Productivity costs per hour is based on the average hourly wage in the Netherlands, which is estimated to be €31.60 for women and €37.90 for men in 2014€ (Hakkaart-van Roijen et al., 2016), leading to €34.18 for women and €40.99 for men when indexed to 2020€.

Presenteeism was measured by asking parents how many hours they would have had to work in order to make up for the time they were hindered by the behavioural problems of their child while they were at work. These hours were multiplied by the productivity costs per hours. If parents indicated that they had no paid employment, productivity losses for absenteeism and presenteeism were assumed to be zero euro.

For unpaid work, parents were asked how many hours others (family members, other unpaid persons, home care, and other paid persons) had to take over housekeeping tasks due to the behavioural problems of their child. The total number of hours were multiplied by the reference prices for productivity loss of unpaid work, which is €14.00 in in 2014€. (Hakkaart-van Roijen et al., 2016) and €15.14 in 2020€. Parents that indicated no housekeeping tasks had to be taken over were assumed to have productivity loss for unpaid work of zero euro.

| **Table S1.** Unit cost per resource and costs of productivity loss | | |
| --- | --- | --- |
| **Variable** | **Unit cost (2020 €)** | **Source** |
| ***Resource*** |  |  |
| Psychiatrist/psychologist | 102.15 | CM |
| Social Psychiatric Nurse/Youth healthcare nurse | 77.27 | IYCM |
| General practitioner | 71.38 | CM, Double consult |
| Speech therapist | 32.45 | CM |
| Alternative healer | 58.69 | IYCM |
| Physiotherapist/School doctor/Occupational physician/GP for parent | 35.69 | CM |
| Remedial teacher | 61.04 | IYCM |
| Paediatrician | 69.22 | CM |
| Medical specialist | 56.24 | CM |
| Emergency care | 280.13 | CM |
| Social worker/Neighbourhood team, Family guardian, Child abuse hotline | 70.30 | CM, social work |
| Foster care | 19.19 | IYCM |
| Residential institution | 179.50 | IYCM, crisis care |
| Day or part-time treatment | 183.07 | IYCM |
| University Medical Centre | 694.38 | CM |
| General hospital | 479.14 | CM |
| Psychotherapeutic institution/Psychiatric hospital | 327.03 | CM |
| Babysitter/Pre-, between-, after school day care | 6.96 | IYCM |
| Medical day care/Boddaert Centre (after school day treatment) | 6.96 ^a^ | IYCM, based on reference price for Babysitting/Day Care |
| Addiction treatment | 205.65 | IYCM |
| Child protective services /Lawyer/Court/Probation | 117.38 | IYCM |
| Police/Bureau Halt (alternative to criminal procedure) | 77.27 | IYCM |
| Truancy officer | 45.46 | D |
| Juvenile detention centre | 598.65 | IYCM |
| ***Medication for child*** | | |
| Ritalin  Tablet 10mg (Brocacef b.v.) | 0.11 | M |
| Methylphenidate  HCL Mylan tablet 10mg (Mylan b.v.) | 0.05 | M |
| Concerta  Tablet MVA 18mg (Brocacef b.v.) | 0.79 | M |
| Equasym  XL Capsule MGA 10mg (Medcor specials b.v.) | 0.62 | M |
| Medikinet  Tablet 10mg (Bmodesto b.v.) | 0.11 | M |
| Dexamfetamine  Sulfaat capsule 2.5mg FNA MR (Fna-voorschriften p/o knmp) | 0.09 | M |
| Strattera  Capsule 25mg (Pluripharm distrimed bv) | 2.69 | M |
| Dipirperon  Tablet 40mg (Eumedica s.a.) | 0.06 | M |
| Pipamperon  Tablet 10mg ACE (Ace apotheek) | 0.19 | M |
| Risperdal  Tablet omhuld 0.5mg (Janssen-cilag b.v.) | 0.18 | M |
| Risperidone  Accord tablet omhuld 4mg (Accord healthcare) | 0.05 | M |
| ***Productivity costs parent*** | | |
| Productivity costs paid employment women | 34.18 | CM |
| Productivity costs paid employment men | 40.99 | CM |
| Productivity costs unpaid work | 15.14 | CM |
| Sources:  CM (Cost Manual, Hakkaart-van Roijen et al., 2016)  IYCM (Intensive Youth Care Manual, Bouwmans et al., 2012)  M (medicijnkosten.nl, last accessed on September 15, 2022).  D (Drost et al., 2012)  ^a^ Reference costs for these services could not be found. We have assumed that the costs of these services are equal to those of Babysitting/Day care, however, it is likely that these costs are higher in reality as children often receive treatment or care in these facilities. | | |

**Appendix S2. Missing data**

We used multiple imputation to handle missing data. An overview of frequencies and percentages of missing variables can be found in Table S2.

| **Table S2: frequency and percentage missing** | | |
| --- | --- | --- |
|  | Missing at T0 (baseline)  N=209 | Missing at T2  N=209 |
| Age of child | 10 (4.78%) |  |
| Age of parent | 13 (6.22%) |  |
| ADHD symptom severity | 1 (0.48%) |  |
| ODD symptom severity | 1 (0.48%) |  |
| Impairment | 1 (0.48%) | 1 (0.48%) |
| EQ5D | 1 (0.48%) | 1 (0.48%) |
| Healthcare cost child | 43 (20.57%) | 31 (14.83%) |
| Other services child | 18 (8.61%) | 24 (11.48%) |
| Medication child | 8 (3.83%) | 11 (5.26%) |
| Healthcare cost parent | 17 (8.13%) | 18 (8.61%) |
| Other services parent | 11 (5.26%) | 6 (2.87%) |
| Absenteeism | 4 (1.91%) | 6 (2.87%) |
| Presenteeism | 1 (0.48%) | 3 (1.44%) |
| Unpaid work costs | 5 (2.39%) | 8 (3.83%) |
| **Note:** only variables with missing values used in the analyses are displayed. | | |

**Appendix S3. Sensitivity Analyses: Methods**

We performed several sensitivity analyses to verify the robustness of our results. First, since we only observed the last 3 months of healthcare and support service utilisation of an (approximate) 12-month period, it is possible that we did not observe the healthcare utilisation caused by the treatment. It is likely that the majority of utilisation occurs in the months after the child is registered with the institution. Therefore, we included extra costs for each of the treatments (i.e., parent training, psychoeducation, child therapy, diet treatment, neurofeedback, cognitive training, family-focused treatment, creative therapy, and social skills training) for which parents indicated that they had started at T1 and/or T2. It was not possible to include the other treatment category, as it was often unclear what the treatment exactly consisted of. The extra costs were based on an assumed number of sessions (often a range) as recommended by guidelines or which were found on websites of practitioners, and therefore assumed to be used in clinical practice, multiplied by the reference prices of a consult with a psychologist/psychiatrist/psychotherapist or a social psychiatric nurse/youth health care nurse, depending on the type of treatment. The numbers used for the sensitivity analyses can be found in Table S5A. The extra costs were added after multiple imputation and applied in four different cases: 1) assuming a low number of sessions; 2) assuming a high number of sessions; 3) reducing the costs of a low number of sessions by 10%, and 4) increasing the costs of a higher number of sessions by 10%.

Additionally, as assumptions were made in the derivation of (health) service and medication utilisation and therefore cost, two sensitivity analyses were performed in which the total costs for (health) services and medication are increased and decreased by 10%. In sensitivity analysis 5 and 6, 10% of total (health) service and medication costs were added and deducted, respectively, in each imputed dataset after multiple imputation and before log transformations of costs.

Since the results of propensity score matching can be sensitive to the matching method chosen, we used an alternative propensity score method to estimate our results in sensitivity analysis 7. We employed inverse probability of treatment weighting as an alternative propensity score method, which uses weights equal to the inverse of the probability of receiving the treatment to estimate weighted mean differences (Austin, 2011b).

We performed three sensitivity analyses in which we dropped specific participants from the sample. These participants were dropped from the sample after multiple imputation, but before propensity score matching. In sensitivity analysis 8, we excluded children diagnosed with autism spectrum disorder (ASD) at T1 and/or T2. When a child is diagnosed with ASD, the problem that leads to the greatest impairment in functioning has to be treated according to the Federation of Medical Specialists (2008). ASD diagnosis could therefore possibly lead to a different treatment recommendation than those for children with ADHD and/or behavioural problems.

A small group of parents in the treatment without medication group did not indicate for any of the non-medication treatments that these had started at T1 or T2, even though some did indicate that they had decided to take some of those treatments. It may be the case that children who do not start any treatment experience worsened symptom severity or impairment due to lack of treatment. On the other hand, it is also possible that children without treatment may not have required any treatment due to low symptom severity or impairment. Either of the possible options may influence the results. In sensitivity analysis 9, we performed the main analysis excluding these children from the reference group.

Finally, we excluded children who had received treatment in the year prior to T0 from the sample in sensitivity analysis 10. Having received treatment previously, either in the form of medication or non-medication interventions, could lead to reduced impact of the observed treatments compared to when a child receives treatment for the first time. This could lead to underestimation of the treatment effect for the latter group.

| **Table S3: Assumed number of sessions and reference prices in sensitivity analyses** | | | | | | | |
| --- | --- | --- | --- | --- | --- | --- | --- |
| **Intervention** | | **Number of sessions** | **Costs (2020€)** | **Low sessions * costs (2020€)** | **High session * costs (2020€)** | **Costs low number of sessions - 10%** | **Costs high number of sessions + 10%** |
| **Behavioural parent training** |  | 12 ^a^ | 102.15 ^k^ | 1225.80 | 1225.80 | 1103.22 | 1348.38 |
| **Psychoeducation** | If a specific program is not mentioned | 1 ^b^ | 102.15 ^k^ | 102.15 | 102.15 | 91.94 | 112.37 |
|  | *Ik ben speciaal training* | 9-15 ^c^ | 102.15 ^k^ | 919.35 | 1532.25 | 827.42 | 1685.48 |
| **Child therapy** |  | 5-10 ^d^ | 102.15 ^k^ | 510.75 | 1021.50 | 459.68 | 1123.65 |
| **Diet treatment** |  | 6 ^e^ | 77.27 ^l^ | 463.62 | 463.62 | 417.26 | 509.98 |
| **Neurofeedback** |  | 25-50 ^f^ | 77.27 ^l^ | 1931.75 | 3863.50 | 1738.58 | 4249.85 |
| **Cognitive training** |  | 25-31 ^g^ | 77.27 ^l^ | 1931.75 | 2395.37 | 1738.58 | 2634.91 |
| **Family treatment** |  | 11-48 ^h^ | 77.27 ^l^ | 849.97 | 3708.96 | 764.98 | 4079.86 |
| **Creative therapy** |  | 6-24 ^i^ | 77.27 ^l^ | 463.62 | 1854.48 | 417.26 | 2039.93 |
| **Social skills training** |  | 36 ^j^ | 77.27 ^l^ | 2781.72 | 2781.72 | 2503.55 | 3059.89 |
| Sources and notes:  ^a^ Federation of Medical Specialists (2008).). ^b^ We assume that children and parents receive at least 1 session of psychoeducation. ^c^ Kenniscentrum Kinder- en Jeugdpsychiatrie( n.d.). ^d^ Praktijk voor Kinder- en Jeugdtherapie (n.d.). ^e^ (Pelsser RED Centrum, n.d.) Based on the information on the web page of Pelsser RED Centrum, we consider 6 sessions with parent and/or child. The preparation work that is also listed are not included in the number of sessions. ^f^ Federation of Medical Specialists, (2018). ^g^ van Berkel (2019) and Prins (2017). ^h^ CJG Rijnmond (n.d) and Karakter Kinder-en Jeugdpsychiatrie (n.d.). ^i^ GGZ Standaarden (2017). ^j^  van de Wiel et al. (2014). ^k^ Reference price for a session with a psychologist or psychiatrist (Hakkaart-van Roijen et al., 2016). ^l^  Reference price for a session with a social psychiatric nurse (Bouwmans et al., 2012). | | | | | | | |

**Appendix S4. Attrition**

We compared baseline characteristics of individuals who had (partially) completed all three surveys (pattern 1) to those who had either (partially) completed survey 1 only (pattern 2, n=113, 30.7%) or survey 1 and 2 only (pattern 3, n=35, 9.5%) using Welch’s t-tests and chi-squared tests. The results can be found in Table S4A and S4B. A comparison between individuals in pattern 1 and pattern 2 showed that individuals who had completed all three surveys reported significantly higher ADHD symptom severity for their child than those who had only completed T0. Similarly, we found that individuals who had completed all three surveys reported significantly higher ADHD symptom severity as well as ODD symptom severity for their child than those who had filled out only T0 and T1 surveys. Possible explanations for this include that those who dropped out of the survey were those parents to children for whom short treatment or no treatment was sufficient, as they had lower symptom severity on average, or that these families were unable or unwilling to further participate in the study or treatment for other unobserved reasons, such as organizational capabilities.

| **Table 4: Sample Attrition: Comparison of baseline characteristics**  **Table S4A Questionnaire T0 and T1 only vs questionnaire T0, T1 and T2** | | | | |
| --- | --- | --- | --- | --- |
| Variable | Pattern 111  N=209 | Pattern 11.  N=35 | Mean difference | P-values |
| Age of child | 8.28  N=199 | 8.53  N=32 | 0.25 | 0.4097 |
| Age of parent | 37.97  N=196 | 39.41  N=34 | 1.44 | 0.3226 |
| ADHD symptom severity | 1.60  N=208 | 1.31  N=34 | -0.29 | 0.0114 |
| ODD symptom severity | 1.29  N=208 | 0.95  N=34 | -0.34 | 0.0175 |
| Impairment | 3.27  N=208 | 2.80  N=32 | -0.48 | 0.1041 |
| MHI-5 parent | 72.48  N=209 | 76.11  N=35 | 3.64 | 0.1495 |
| Parental stress | 44.00  N=208 | 43.41  N=32 | -0.59 | 0.7021 |
| Social support sum | 65.63  N=209 | 65.71  N=35 | 0.08 | 0.9737 |
| EQ5D | 0.78  N=208 | 0.81  N=32 | 0.03 | 0.2337 |
| Sex of child (female) | 60  N=209 | 10  N=35 |  | 0.987 |
| Sex of parent (female) | 197  N=209 | 12  N=35 |  | 0.995 |
| Treatment history | 52  N=209 | 10  N=35 |  | 0.642 |
| Self-reported medication treatment history | 22  N=209 | 6  N=35 |  | 0.256 |
| Note: pre-imputed data. Welch’s t-test and chi-square test. Variable means and mean differences are rounded up. | | | | |

| **Table 4: Sample Attrition: Comparison of baseline characteristics**  **Table S4B: Questionnaire T0 only vs questionnaire T0, T1, and T2** | | | | |
| --- | --- | --- | --- | --- |
| Variable | Pattern 111  N=209 | Pattern 1..  N=113 | Mean difference | P-values |
| Age of child | 8.28  N=199 | 8.40  N=100 | 0.12 | 0.6012 |
| Age of parent | 37.97  N=196 | 38.47  N=99 | 0.50 | 0.4907 |
| ADHD symptom severity | 1.60  N=208 | 1.40  N=103 | -0.21 | 0.0036 |
| ODD symptom severity | 1.29  N=208 | 1.16  N=103 | -0.13 | 0.1292 |
| Impairment | 3.27  N=208 | 3.29  N=33 | 0.01 | 0.9496 |
| MHI-5 parent | 72.48  N=209 | 74.55  N=110 | 2.07 | 0.2268 |
| Parental stress | 44.00  N=208 | 43.28  N=36 | -0.72 | 0.5777 |
| Social support sum | 65.63  N=209 | 65.34  N=109 | -0.29 | 0.8343 |
| EQ5D | 0.78  N=208 | 0.79  N=33 | 0.01 | 0.7702 |
| Sex of child (female) | 60  N=209 | 26  N=103 |  | 0.519 |
| Sex of parent (female) | 197  N=209 | 105  N=113 |  | 0.635 |
| Treatment history | 52  N=209 | 19  N=100 |  | 0.250 |
| Self-reported medication treatment history | 22  N=209 | 7  N=100 |  | 0.320 |
| Note: pre-imputed data. Welch’s t-test and chi-square test. Variable means and mean differences are rounded up. | | | | |

**Appendix S5. Treatment combinations**

Children in our sample often received non-medication treatments. Table S5 shows the frequencies and percentages with which various non-medication treatments were reported to have been started during the care trajectory.

| **Table S5: Treatment combinations in raw data** | | | |
| --- | --- | --- | --- |
| Intervention type ^a^ | Treatment with medication  (n=108) | Treatment without medication  (n=101) | Total  (n=209) |
|  | Frequency (%) ^b^ | Frequency (%) ^b^ | Frequency (%) ^b^ |
| Medication | 108 (100.00%) | 0 (0.00%) | 108 (51.68%) |
| Behavioural parent training | 68 (62.96%) | 43 (42.57%) | 111 (53.11%) |
| Psychoeducation | 66 (61.11%) | 23 (22.77%) | 89 (42.58%) |
| Child therapy | 55 (50.93%) | 39 (38.61%) | 94 (44.98%) |
| Diet treatment | 1 (0.93%) | 2 (1.98%) | 3 (1.44%) |
| Neurofeedback | 5 (4.63%) | 0 (0.00%) | 5 (2.39%) |
| Cognitive training | 16 (14.81%) | 6 (5.94%) | 22 (10.53%) |
| Family treatment | 4 (3.70%) | 3 (2.97%) | 7 (3.35%) |
| Creative therapy | 3 (2.78%) | 5 (4.95%) | 8 (3.83%) |
| Social skills training | 1 (0.93%) | 2 (1.98%) | 3 (1.44%) |
| Other treatments ^c^ | 5 (4.63%) | 5 (4.95%) | 10 (4.78%) |
| Notes:  ^a^ A child-parent pair is said to have started the intervention if the parent indicated at T2 and/or T3 that the intervention has either started and 1) is ongoing; 2) has ended prematurely; or 3) has been completed.  ^b^ Percentages can exceed 100% as child-parent pairs can start multiple treatments.  ^c^ Parent responses indicate that some type of intervention happened, but 1) it is unclear what the intervention was (e.g., because the parent only provided the name of the care provider); or 2) the intervention is aimed at something other than externalising behaviours (such as tics, language development disorder, trauma, or ASD). For 3 parents responses to started treatment was missing at T3, therefore their treatment assignment was determined only responses at T2.  This group also included treatments that were clearly not intended to treat externalising behaviours (e.g., Eye Movement Desensitization and Reprocessing [EMDR] or Pivotal Response Treatment [PRT]) and children for whom it was unclear which non-medication treatment had been provided (e.g., when parents provided the name of care providers rather than treatments).  Treatment with medication: children with reported medication treatment initiation alone or in combination with non-medication treatments.  Treatment without medication: children with no reported medication treatment initiation. | | | |

**Appendix S6. Sensitivity Analyses: Results**

We conducted several sensitivity analyses to test the robustness of our effects under the assumptions that were made in our main analysis. The results of the sensitivity analyses can be found in Table S5B. Results were not sensitive to the exclusion of children with reported ASD diagnoses or the exclusion of children who had not started any treatment at T1 and/or T2. When we used inverse probability weighting as a propensity score method, the incremental effectiveness estimates for QALYs, ADHD and ODD symptom severity and impairment had 95%-CIs that included zero, but the incremental cost estimates were positive and had 95%-CIs that did not include zero. Incremental cost estimates were sensitive to the exclusion of those who reported treatment history in the previous year and some assumptions regarding costs which led to incremental cost estimates that were positive and had 95%-CIs that excluded zero. The latter may be due to the fact that children in the treatment with medication group have higher percentages of reporting treatment for most of the treatments than those in the treatment without medication group, which means that the treatment with medication group has more costs added in these sensitivity analyses, thereby increasing the difference in costs between the groups. Indeed, in sensitivity analyses where we increased and decreased total health and other support service costs by 10%, the difference in costs remained insignificant.

| **Table S6: Sensitivity Analyses: interpolated costs and effects** | | | | | |
| --- | --- | --- | --- | --- | --- |
| **Outcomes**  **Sensitivity Analysis** | **QALY** | **ADHD symptom severity** | **ODD symptom severity** | **Impairment** | **Total costs** |
| **Main analysis** | 0.005  (-0.048; 0.063) | -0.057  (-0.234; 0.113) | -0.100  (-0.265; 0.127) | 0.149  (-0.342; 0.620) | 389.23  (-16.48; 809.23 |
| 1. **Additional costs: Low number of sessions** |  |  |  |  | 1614.44  (239.65; 3093.98) |
| 1. **Additional costs: High number of sessions** |  |  |  |  | 1910.94  (262.26; 3663.14) |
| 1. **Additional costs: Low number of sessions – 10%** |  |  |  |  | 1485.62  (194.10; 2875.40) |
| 1. **Additional costs: High number of sessions + 10%** |  |  |  |  | 2065.39  (311.98; 3924.40) |
| 1. **10% increased health and other service costs** |  |  |  |  | 431.53  (-13.48; 892.21) |
| 1. **10% decreased health and other service costs** |  |  |  |  | 347.23  (-19.00; 726.29) |
| 1. **Alternative propensity method: IPTW** | 0.018(-0.026; 0.067) | -0.093(-0.238; 0.034) | -0.050(-0.233; 0.090) | 0.047(-0.350; 0.422) | 362.93(22.45; 737.96) |
| 1. **Excluding ASD** | 0.025  (-0.027; 0.092) | -0.100  (-0.379; 0.068) | -0.098  (-0.336; 0.119) | 0.021  (-0.565; 0.589) | 176.93  (-346.83; 782.60) |
| 1. **Excluding those not treated** | 0.017  (-0.057; 0.073) | -0.062  (-0.286; 0.138) | -0.011  (-0.223; 0.236) | 0.144  (-0.405; 0.730) | 395.98  (-78.92; 919.21) |
| 1. **Excluding treatment history** | 0.004  (-0.074; 0.062) | -0.069  (-0.271; 0.164) | -0.065  (-0.271; 0.190) | 0.154  (-0.319; 0.829) | 496.45  (27.44; 1037.07) |
| Note: all bootstrapped percentile 95%-CI’s are based on 25*400 = 10,000 replications. No changes were made to the sample in sensitivity analyses 1-6, therefore the results for the effectiveness outcomes are the same as in the main analysis.  *Treatment with medication: children with reported medication treatment initiation alone or in combination with non-medication treatments.*  *Treatment without medication: children with no reported medication treatment initiation.* | | | | | |

| **Table S6. Continued.** | | | | |
| --- | --- | --- | --- | --- |
| **Outcomes**  **Sensitivity Analysis** | ***ICER***  ***QALY*** | ***ICER***  ***ADHD symptom severity*** | ***ICER***  ***ODD symptom severity*** | ***ICER Impairment*** |
| **Main analysis** | 77,354.01 | -6,779.43 | -3,876.39 | 2,617.53 |
| 1. **Additional costs: Low number of sessions** | 320,846.16 | -28,119.46 | -16,078.36 | 10,856.90 |
| 1. **Additional costs: High number of sessions** | 379,771.19 | -33,283.73 | -19,031.23 | 12,850.83 |
| 1. **Additional costs: Low number of sessions – 10%** | 295,245.69 | -25,875.79 | -14,795.46 | 9,990.63 |
| 1. **Additional costs: High number of sessions + 10%** | 410,466.41 | -35,973.91 | -20,569.44 | 13,889.50 |
| 1. **10% increased health and other service costs** | 85,759.27 | -7,516.08 | -4,297.60 | 2,901.95 |
| 1. **10% decreased health and other service costs** | 69,007.43 | -6047,92 | -3,458.13 | 2,335.10 |
| 1. **Alternative propensity method: IPTW** | 20,167.33 | -3,912.32 | -7,249.85 | 7,657.87 |
| 1. **Excluding ASD** | 7,093.05 | -1,766.19 | -1,806.79 | 8,653.02 |
| 1. **Excluding those not treated** | 23,539.58 | -6,435.51 | -35,304.11 | 2,758.19 |
| 1. **Excluding treatment history** | 125,508.40 | -7,167.79 | -7,670.98 | 3,219.00 |

**References**

Austin, P. C. (2011). An introduction to propensity score methods for reducing the effects of

confounding in observational studies. *Multivariate Behavioral Research, 46(3),* 399–424.

Bouwmans, C. A. M., Schawo, S. J., Jansen, D. E. M. C., Vermeulen, K. M., Reijneveld, S. A., &

Hakkaart-Van Roijen, L. (2012). *Handleiding Vragenlijst Intensieve Jeugdzorg: Zorggebruik en productieverlies.*

CBS (2021). *Consumentenprijzen; prijsindex 2015=100*. Available from:

<https://opendata.cbs.nl/#/CBS/nl/dataset/83131NED/table> [Accessed on: 19 December

2022].

CJG Rijnmond (n.d.). *Stop! Hoe los ik het op?* Retrieved January 3, 2023 from

https://cjgrijnmond.nl/cursus/bar-stop-hoe-los-ik-het-op.

Drost, R.M.W.A., Paulus, A.T.G., Ruwaard, D., & Evers, S.M.A.A. (2014). *Handleiding intersectorale*

*kosten en baten van (preventieve) interventies: Classificatie, Identificatie en Kostprijzen*.

Dutch National Health Care Institute (Zorginstituut Nederland). (2022). *Medicijnkosten.nl.*

<https://www.medicijnkosten.nl/> [Accessed on 15 September 2022].

Federation of Medical Specialists (Federatie Medisch Specialisten) (2008). *Autismespectrumstoornis*

*bij kinderen/jeugd.*

Federation of Medical Specialists (Federatie Medisch Specialisten). (2018). *ADHD bij kinderen.*

GGZ Standaarden (2017). *Generieke module Vaktherapie*.

Hakkaart-van Roijen, L., van der Linden, N., Bouwmans, C., Kanters, T., & Swan Tan, S. (2016).

*Kostenhandleiding: Methodologie van kostenonderzoek en referentieprijzen voor economische evaluaties in de gezondheidszorg*.

Karakter Kinder- en Jeugdpsychiatrie (n.d.). *Intensieve Psychiatrische Gezinsbehandeling (IPG).*

Retrieved January 3, 2023 from https://www.karakter.com/behandelingen/ipg.

Kenniscentrum Kinder- en Jeugdpsychiatrie (n.d.). *Ik ben Speciaal*. Retrieved January 3, 2023 from

https://www.kenniscentrum-kjp.nl/professionals/behandelmethoden/ik-ben-speciaal/.

Koopmanschap, M.A., Rutten F.F., van Ineveld, B.M., van Roijen, L. (1995). The friction cost method

for measuring indirect costs of disease. *Journal of Health Economics*. 14(2): 171-89

Pelsser RED Centrum (n.d.). *Werkwijze en kosten – Pelsser RED Centrum*. Retrieved January 3, 2023

from https://www.adhdenvoeding.nl/werkwijze-en-kosten/.

Praktijk voor Kinder- en Jeugdtherapie (n.d.). *Praktijk voor kinder- en jeugdtherapie: Veelgestelde*

*vragen.* Retrieved January 3, 2023 from https://www.kinderenjeugdtherapie.com/de-praktijk/veelgestelde-vragen/.

Prins, P. (2017). *Databank effectieve jeugdinterventies: beschrijving ‘Braingame Brian: een executieve*

*functietraining met game-elementen voor kinderen met ADHD en executieve*

*functieproblemen’.* Nederlands Jeugdinstituut.

van Berkel, S. (2019). *Databank effectieve jeugdinterventies: beschrijving ‘Cogmed*

*Werkgeheugentraining’. Nederlands Jeugdinstituut.*

Van de Wiel, N., Hoppe, A. & Matthys, W. (2014*). Databank effectieve jeugdinterventies: beschrijving*

*‘Minder boos en opstandig’.* Nederlands Jeugdinstituut.

van den Hoofdakker, B. & van der Veen-Mulders, L. (2018). *Databank effectieve jeugdinterventies:*

*beschrijving ‘BPTG-G voor kinderen met ADHD en gedragsproblemen’*. Nederlands Jeugdinstituut.

1. None of the parents in the sample indicated Risperdal Consta use, therefore it was not included in the calculation of total costs. [↑](#footnote-ref-1)
2. I.e. $dose*\left( price per{tablet}/{mg in the tablet} \right)*frequency per day*number of days$ [↑](#footnote-ref-2)
